# Solitary Pulmonary Nodule: Morphological Effects on Metabolic Activity Assessment

**DOI:** 10.4274/mirt.galenos.2019.65707

**Published:** 2019-09-06

**Authors:** Mehmet Erdoğan, Şehnaz Evrimler, Hüseyin Aydın, Adnan Karaibrahimoğlu, Sevim Süreyya Şengül

**Affiliations:** 1 Süleyman Demirel University Faculty of Medicine, Department of Nuclear Medicine, Isparta, Turkey; 2 Süleyman Demirel University Faculty of Medicine, Department of Radiology, Isparta, Turkey; 3 Süleyman Demirel University Faculty of Medicine, Department of Biostatistics and Medical Informatics, Isparta, Turkey

**Keywords:** Positron emission tomography, solitary pulmonary nodule, metabolic tumor volume, total lesion glycolysis

## Abstract

**Objectives:**

We aimed to evaluate the effects of morphological characteristics of the solitary pulmonary nodules (SPN) on metabolic activity assessment. To the best of our knowledge, this is the first study to compare the volumetric metabolic activity parameters according to the morphologic parameters of the nodules.

**Methods:**

In this retrospective study, ^18^F-FDG positron emission tomography and computed tomography scans performed between 2011 and 2018 were evaluated by a nuclear and diagnostic radiologist. One hundred thirteen patients with SPNs with biopsy-proven diagnosis were included. SPNs were classified as solid, partially solid (PS), and ground glass opacity (GGO).

**Results:**

SPN diameter, SUV_max_, metabolic tumor volume (MTV), total lesion glycolysis (TLG), and density were significantly higher in the malignant group. SUV_max_, MTV, TLG increased in direct proportion to the diameter. There was no a significant difference between GGO, PS, and solid nodules in terms of SUV_max_ values. MTV and TLG values increased in parallel with the density of the nodules, but this increase was only significant in the malignant group. There was a significant difference between SPNs <2 cm and SPNs ≥2 cm in terms of MTV, while there was no difference in terms of SUV_max_. The cut-off value determined by the ROC curve was found to be 4.39 for SUV_max_, 7.33 mL for MTV and 31.88 g for TLG. The cut-off values for SUV_max_ of solid and subsolid nodules were close to each other, but cut-off values for MTV and TLG were higher in solid nodules.

**Conclusion:**

SUV_max_, MTV, and TLG are affected by diameter and attenuation. We suggest using different MTV and TLG cut-off values for solid and subsolid nodules, but we suggest using same SUV_max_ values. MTV can be a more reliable parameter than SUV_max_ in prediction of malignancy in smaller nodules.

## Abstract (tr)

**Amaç:**

Soliter pulmoner nodüllerin (SPN) morfolojik özelliklerinin metabolik aktivite değerlendirmesi üzerindeki etkilerini değerlendirmeyi amaçladık. Bildiğimiz kadarıyla, volümetrik metabolik aktivite parametrelerini nodüllerin morfolojik parametrelerine göre karşılaştıran ilk çalışma budur.

**Yöntem:**

2011 ve 2018 yılları arasında yapılan ^18^F-FDG pozitron emisyon tomografisi ve bilgisayarlı tomografi taramaları, bir nükleer tıp uzmanı ve radyoloji uzmanı tarafından retrospektif olarak değerlendirildi. Biyopsi ile kanıtlanmış tanısı olan 113 SPN hastası dahil edildi. SPN’ler solid, kısmi solid (PS) ve buzlu cam opasitesi (GGO) olarak sınıflandırıldı

**Bulgular:**

SPN çapı, SUV_maks_, metabolik tümör hacmi (MTV), toplam lezyon glikoliz (TLG) ve dansite malign grupta anlamlı olarak yüksek bulundu. SUV_maks_, MTV ve TLG değerleri çap ile doğru orantılı olarak arttı. GGO, PS ve solid nodüller arasında SUV_maks_ değerleri arasında anlamlı bir fark yoktu. MTV, TLG değerleri nodüllerin yoğunluğu ile paralel olarak arttı; ancak sadece malign grupta anlamlı fark bulundu. 2 cm’den küçük grupta MTV için anlamlı fark varken SUV_maks_ için yoktu. ROC eğrisi ile belirlenen kesme değerinin SUV_maks_ için 4,39, MTV için 7,33 mL, TLG için 31,88 g olduğu bulundu. Solid ve subsolid nodüllerin SUV_maks_ için cut-off değeri birbirine yakındı, ancak MTV, TLG için cut-off değer solid nodüllerde daha yüksekti.

**Sonuç:**

SUV_maks_, MTV ve TLG çap ve atenüasyondan etkilenmektedir. Solid ve subsolid nodüller için farklı MTV ve TLG cut-off değerlerinin kullanılmasının gerektiğini; ancak SUV_maks_ için gerekli olmadığını düşünmekteyiz. MTV, küçük nodüller için malignite tahmininde SUV_maks_’tan daha güvenilir bir parametre olabilir.

## Introduction

A solitary pulmonary nodule (SPN) is a well-defined, round or oval lesion with a diameter less than 3 cm, surrounded by normal parenchyma, not associated with atelectasis, lymphadenopathy, pneumonia and pleural effusion (1,2). SPNs are detected in 0.9-2% of chest X-rays and 90% of them are seen incidentally (3). Multidetector computed tomography (MDCT) allows detection of nodules with smaller sizes, even with diameter of 1-5 mm, which results in increase in detection rates of SPNs ranging between 8-51% (4,5). SPNs are classified as solid and subsolid nodules. Subsolid nodules can be either pure ground glass opacity (GGO) or semisolid (SS). The etiology of SPNs can be benign or malignant. If MDCT and follow-up imaging findings are indeterminate, ^18^F-FDG positron emission tomography (PET)/CT or biopsy is needed for precise diagnosis (6,7). ^18^F-FDG PET/CT is a non-invasive technique that demonstrates the amount of glucose metabolism used by metabolically active cells, gives morphological information and provides differentiation between malignant and benign lesions (8). A standardized uptake value (SUV) is a semiquantitative method for evaluation of^ 18^F-FDG uptake besides qualitative interpretation with the PET scans. The maximum SUV (SUV_max_) >2.5 is accepted as a threshold value for malignant lesions, although there can be some variations in the literature (8,9,10,11). On the other hand, ^18^F-FDG avidity can also be observed in benign conditions such as inflammation, infection; or malignant diseases can be less avid secondary to volumetric effects such as nodule size (12,13,14,15,16,17,18,19). Kim et al. (20) has mentioned that, SUV_max_ values of half of the bronchoalveolar carcinoma and carcinoid tumors, which constitute 2% of all lung cancers, cause false negative PET results.

Metabolic activity of the lesions can be measured with volumetric parameters, metabolic tumor volume (MTV) and total lesion glycolysis (TLG). The MTV is a volumetric measurement of tumor cells measured by semi-automatic delineation tools using a specific threshold of SUV. TLG is defined as the product of the mean SUV and the MTV. SUV_mean_ is the mean value of SUV in a chosen region (21).

The aim of this study was to evaluate morphological and metabolic activity parameters for SPNs and the effects of morphological characteristics of the nodule on metabolic activity assessment. To the best of our knowledge, this is the first study to compare the volumetric metabolic activity parameters according to the morphologic parameters of the nodules.

## Materials and Methods

^18^F-FDG PET/CT scans were performed in a Lutetium-Yttrium Oxyorthosilicate (LYSO) PET/64-slice CT scanner (Philips Gemini TF) for pulmonary nodule assessment between 2011 and 2018. PET/CT images were evaluated retrospectively by a nuclear radiologist with 8 years of experience (M.E.). Approval from the local research ethics committee was granted. One hundred thirteen SPNs with biopsy-proven diagnosis were included in the study. Histopathological findings were accepted as the gold standard method. Patients with multiple nodules, and calcified nodules were excluded.

Non-contrast Thorax CT scans were performed with Siemens Somatom Definition AS, 128 slice CT machine. Imaging parameters were as follows; automatic effective mA, 120 kVp, gantry rotation speed 0.5 sec, slice thickness 1 mm. Images were retrospectively evaluated by a diagnostic radiologist with 15 years of experience (H.A). The radiologist was blinded to the histopathology and PET findings. Nodule size, location, margins, density, vascular sign, and pleural tag were evaluated individually. SPNs were classified as solid, SS, GGO nodules according to their densities. Besides, their densities were calculated by region of interest (ROI) replacement in Hounsfield unit (HU).

**Figure 1:**
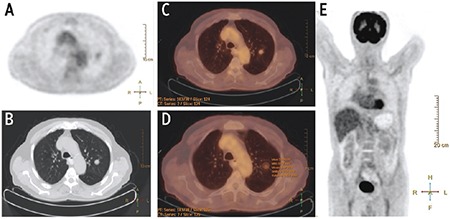
A well-defined and solid solitary pulmonary nodules was detected in left upper lobe in positron emission tomography (PET) image. (A) Axial non-contrast Thorax computed tomography (CT) at parenchyma window (B), and PET/CT image (C). Measurement of activity parameters were consistent with benign lesions on PET/CT image (D). MIP image of total body PET showed no significant activity (E)

**Figure 2:**
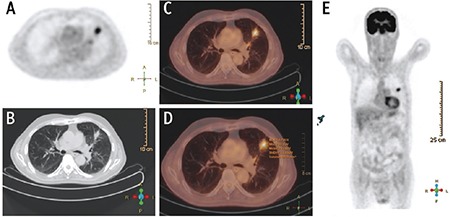
A spiculated and solid solitary pulmonary nodules was detected in left upper lobe in positron emission tomography (PET) image. (A) Axial non-contrast Thorax computed tomography (CT) at parenchyma window (B), and PET/CT image (C). Measurement of activity parameters were consistent with malignancy on PET/CT image (D). MIP image of total body PET showed significant activity in left upper lobe (E)

^18^F-FDG PET/CT scans were performed after 6-hours fastening. Three point seven MBq/kg (0.1 mCi/kg) ^18^F-FDG was given by intravenous injection. PET and CT images (non-corrected and attenuation-corrected) were obtained using maximum intensity projection and cross-sectional methods. SUV_max_, metabolic activity volumetric parameters such as MTV, and TLG were calculated. MTV was calculated by ROI replacement in metabolically active area in each slice. TLG was calculated as the product of SUV_mean_ and MTV (SUV_max_) >2.5 was accepted as a threshold value for malignant lesions (Figure 1, 2).

Both of the diagnostic radiologist and the nuclear radiologist made a final assessment for prediction of benignity or malignancy, independently from each other.

### Statistical Analysis

Statistical analyses of the study were performed by SPSS 20.0 (IBM Inc., Chicago, IL, USA). Descriptive statistics were presented as mean±standard deviation for continuous variables and as frequency (percentage) for categorical variables with tables. Normality of continuous variables were checked by the Kolmogorov-Smirnov test. Mann-Whitney U for two independent groups and Kruskal-Wallis tests for multiple groups were used to compare continuous numerical data since there was no normal distribution. ROC analysis was performed for SUV_max_ and MTV values ​​according to malignancy status and cut-off values were determined. Differential diagnosis rates such as specificity, sensitivity, accuracy and Kappa coefficients were calculated by comparing histopathological, radiological and nuclear medical evaluations. Monte Carlo corrected chi-square analysis was performed to determine the relationship between histopathological tumor subtypes and other categorical variables. P<0.05 was considered as statistically significant result by assuming a type 1 error value of 5% in all analyses.

## Results

The vast majority (79.6%) of patients were male and the average age was 67.88±10.75 years (median=68). According to the histopathological diagnosis, 16.8% (n=19) of SPNs were benign, and 83.2% (n=94) were malignant (Table 1). Malignancy subtypes were adenocarcinoma (37.2%), squamous cell carcinoma (SCC) (25.7%), small cell carcinoma (15.9%), carcinoid tumor (2.7%) and bronchoalveolar carcinoma. Metastasis was detected in one patient. The distribution of SPNs according to attenuation was; solid (77.9%), SS (15.9%) and GGO (6.2%), respectively.

Spiculated margin, vascular sign, and pleural tag presence were predominantly observed in the malignant group (Table 1). The attenuation distribution of nodules was; solid, SS, and GGO, respectively (Table 1). SPN diameter, SUV_max_, MTV, TLG, and density values were significantly different between the malignant and benign SPNs according to the histopathologic results. Those parameters were significantly higher in the malignant group (Table 1, 2). However, no significant difference was found amongst the malignant subtypes.

SUV_max_, MTV, and TLG increased in direct proportion to the SPN diameter (R values were 0.53, 0.70 and 0.75 respectively, p<0.001). When we separated SPNs in two groups according to diameter, such as <2 cm and ≥2 cm, there was a significant difference between groups in terms of MTV (p<0.001), while there was no difference in terms of SUV_max_ (p=0.096) (Table 3). According to margin classification, most of the well-defined ones were SCC, lobulated ones were adenocarcinoma, and spiculated ones were small cell carcinoma and SCC (p=0.036).

According to threshold value of 2.5; the sensitivity, specificity, accuracy, positive predictive value (PPV), and negative predictive value (NPV) of SUV_max_ were found as 98.9%, 52.6%, 91.1%, 91.1%, and 90.9%, respectively (Kappa=0.620). On the other hand, sensitivity, specificity, accuracy, PPV, and NPV of final diagnosis of CT evaluation were calculated as 90.4%, 63.1%, 85.8%, 92.3%, and 57.1%, respectively (Kappa=0.514).

**Figure 3:**
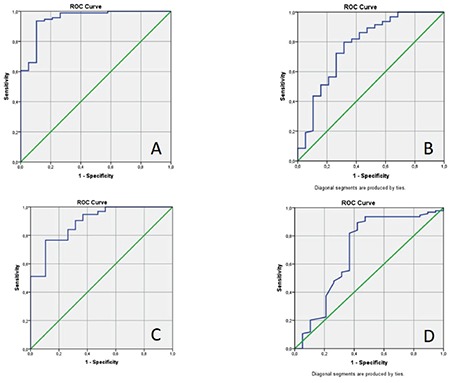
ROC analysis of SUV_max_ (A), metabolic tumor volume (B), total lesion glycolysis (C), and density (D) for malignancy

The cut-off value calculated by the ROC curve analysis for SUV_max_, based on the likelihood of malignancy, was found to be 4.39 (sensitivity 93.6%, specificity 89.5% and accuracy 92.9%), (AUC=0.950±0.027; p<0.001) (Figure 3). Similarly, ROC analysis for MTV measurements calculated the cut-off value as 7.33 (AUC=0.774±0.066; p<0.001) (sensitivity 79.8%, specificity 68.4%, accuracy 77.9%) (Figure 3). The cut-off value calculated for TLG measurements was 31.88 g (AUC=0.891±0.039; p<0.001) (sensitivity 76.6%, specificity 89.5%, accuracy 78.8%) (Figure 3). The cut-off value calculated for the density was 2.5 HU, while the ROC curve was found to be significant (AUC=0.694±0.080; p=0.008) (Figure 3).

In both benign and malignant groups, there was no significant difference between SUV_max_ values amongst GGO, SS and solid nodules. In benign group MTV and TLG values increased in parallel with the density of the nodules, but no significant difference was found. On the other hand, in malignant group, both MTV and TLG values increased in direct proportion to the density of the nodules, significantly (Table 4). Cut-off values of SUV_max_, MTV, TLG for subsolid SPNs were 4.41, 5.22 and 14.06, respectively. Cut-off values of SUV_max_, MTV, and TLG for solid SPNs were 4.39, 17.53 and 73.38, respectively (Table 5).

## Discussion

We investigated the morphological and metabolic activity parameters for SPNs and the effect of morphological characteristics of the nodule on metabolic activity assessment (SUV_max_ and volumetric parameters such as MTV and TLG). In this study, we compared the ^18^F-FDG PET and CT findings of SPNs with histopathological diagnosis of 113 patients. Ninety four of them (83.2%) were malignant and 19 of them (16.8%) were benign.

We accepted SUV_max_>2.5 as a threshold value for malignant nodules and ≤2.5 for benign nodules in ^18^F-FDG PET evaluation, similarly with most of the studies in literature (8,9,10,11). However, ^18^F-FDG avidity can also be observed in benign conditions such as inflammation, infection; or malignant diseases can be less avid secondary to volumetric effects such as nodule size, ROI placement, etc. (12,13,14,15,16,17,18,19). SUV_max_ values of half of the bronchoalveolar carcinoma and carcinoid tumors which constitute 2% of all lung cancers, may cause false negative PET results (20).

In our study, sensitivity, specificity, accuracy, PPV and NPV were 98.9%, 52.6%, 91.1%, 91.1%, and 90.9%, respectively (Kappa=0,620) in comparison with histopathological findings. In a study by Orlacchio et al. (22), the sensitivity, specificity and accuracy were calculated as 76.9%, 100%, and 89.2% according to SUV_max_ threshold of 2.5 in benign (46.4%) and malign (53.6%) SPNs. Opoka et al. (23) calculated 95% sensitivity, 88% specificity, 91.5% accuracy in their study using SUV_max_ threshold value of 2.5 in 40 malignant and 42 benign SPNs. Although we found higher sensitivity, the specificity was lower, and the accuracy was similar in comparison with Orlacchio et al’s. (22) study. We think that, this may result from small patient population in benign group in our study. In addition, 9 of the 19 benign SPNs had infectious etiology in our study. SPNs with infectious, inflammatory, granulomatous etiology can cause higher ^18^F-FDG avidity (11). Deppen et al. (24) found similar sensitivity (92%) and specificity (40%) in their study performed in a region of endemic granulomatous diseases.

Grgic et al. (10) evaluated malignancy ratios of 140 patients with using different SUV_max_ threshold value and found that more than 90% of nodules with SUV_max_<2 were benign. Sensitivity, specificity and NPV were 96%, 55%, and 92%, respectively. The highest diagnostic accuracy was found with SUV_max_ threshold of 4 (sensitivity, specificity and accuracy of 85%). ROC analysis in a study with 88 SPNs by Hou et al. (25) showed SUV_max_>3.635 as the best threshold value for SPNs (sensitivity 83.3%, sensitivity 62.5%, accuracy 79.2%). In our study, ROC analysis demonstrated (AUC=0.950±0.027; p<0.001) SUV_max_ cut-off value as 4.39 for malignant nodules, similar with the study by Grgic et al. (10) and higher than the study by Hou et al. (25) (sensitivity 93.6%, specificity 89.5% and accuracy 92.9%). Sensitivity, specificity and accuracy values for threshold value of 4.39 were also higher than both two studies.

We observed that, SUV_max_ was significantly higher in the malignant group. However, no significant difference was found amongst the malignant histopathologic subtypes. Also, Divisi et al. (12) found no significant correlation between histopathological findings and SUV_max _(p=0.586). Davidson et al. (26) showed that SCC was more ^18^F-FDG avid than adenocarcinoma.

Volumetric parameters developed for measuring metabolic activity are MTV, and TLG in PET scans. In our study, there was a significant difference between malignant and benign nodules in terms of MTV and TLG values. All of the measurements were significantly higher in malignant group. However, there was no significant difference amongst the malignant histopathologic subtypes. ROC analysis showed cut-off value of 7.33 mL for MTV (AUC=0.774±0.066; p<0.001) (sensitivity 79.8%, specificity 68.4%, accuracy 77.9%) and 31.88 g for TLG (AUC=0.891±0.039; p<0.001) (sensitivity 76.6%, specificity 89.5%, accuracy 78.8%). There has been a few studies in literature researching the relationship between volumetric parameters and prognosis in small cell lung cancer, mesothelioma, non-small cell lung cancers (27,28,29). However, according to our literature search, this is the first study to evaluate the volumetric parameters in SPNs.

Winer-Muram (30) has declared that the probability of malignancy increases with the size. Kim et al. (31) observed that SUV_max_ was directly proportional to the lesion size, but inversely proportional to the GGO percentage. Divisi et al. (12) and Khalaf et al. (32) found consistent results with the study by Kim et al. (31) in terms of relation between nodule size and SUV_max_.

We also observed that SPN diameter was significantly larger in the malignant group. In addition, SUV_max_, MTV, TLG increased in direct proportion to the SPN diameter (R values were 0.53, 0.70 and 0.75, respectively, p<0.001). When we separated SPNs according to diameter into two groups, such as <2 cm and ≥2 cm, there was a significant difference between groups in terms of MTV (p<0.001), while there was no difference between groups in terms of SUV_max_ (p=0.096). Therefore, MTV can be a more reliable parameter than SUV_max_ in prediction of malignancy in smaller nodules (p<0.001).

Zhou et al. (33) and Nakamura et al. (34) mentioned that solid component predominancy increases by the invasiveness of adenomatous lung lesions in SS nodules. Our study showed that malignancy rates were directly proportional to the density. On the contrary, there was no significant difference in density measurements of subsolid nodules between benign and malignant group (p=0.70).

Chun et al. (35) revealed that SUV_max_ of SS nodules was higher in benign inflammatory group than in malignant group, but there was no significant difference between groups in terms of GGO nodules. They concluded that follow-up was recommended instead of immediate biopsy for such cases. Nomori et al. (17) evaluated 15 GGO and 101 solid nodules in their study and concluded that ^18^F-FDG PET was not a feasible method for GGO nodules because of having lower sensitivity (10%) and specificity (20%), unlike it was a feasible method for solid nodules because of having higher sensitivity (90%) and specificity (71%). We observed no significant difference amongst GGO, SS and solid nodules neither in benign nor malignant group in terms of SUV_max _values. In both benign and malignant groups, MTV and TLG values increased in parallel with the density of the nodules, but significant difference was only found in malignant group. SUV_max_ cut-off value of solid and subsolid nodules were considerably close to each other, but MTV and TLG were higher in solid nodules than the other groups. Thus, we think that there is a need for using different cut-off levels of MTV and TLG for solid and subsolid nodules, but there is no need for using different cut-off level of SUV_max_.

### Study Limitations

The limitations of our study can be listed as follows; (1) the histopathological findings were results of tru-cut biopsy, not lobectomy, (2) biopsies were performed for only malignancy suspected nodules, others were followed-up radiologically. That was the reason why we had a smaller patient group in the benign group.

## Conclusion

MDCT or ^18^F-FDG PET findings can be indeterminate for malignancy prediction of SPNs on their own, and should be interpreted together. Metabolic activity assessment can be done by measurements of SUV_max_ and volumetric parameters such as MTV and TLG on PET scans. They are all expected to be found higher in malignancy. However, these parameters are affected by morphological characteristics, such as diameter and attenuation of the nodule. According to these differences, it is controversial which parameter is more reliable. We think that, there is a need for using different cut-off levels of MTV and TLG for solid and subsolid nodules, but there is no need for using different cut-off level of SUV_max_. MTV can be a more reliable parameter than SUV_max_ in prediction of malignancy in smaller nodules (<2 cm). We suggest that, further studies are needed for evaluation of volumetric parameters in SPNs.

## Figures and Tables

**Table 1:**
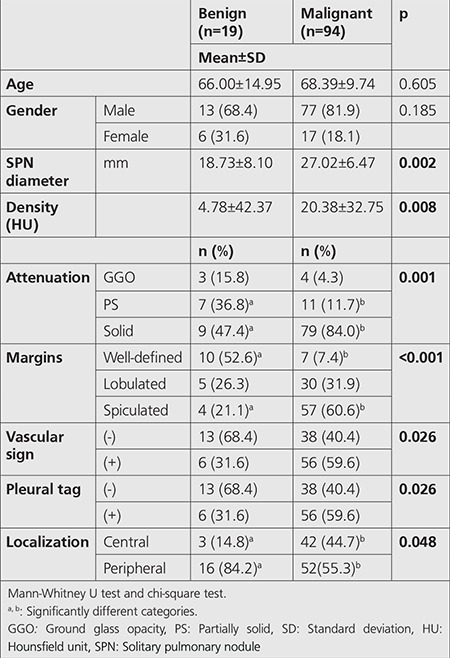
Demographic findings of patients and computed tomography findings of benign-malignant solitary pulmonary nodules

**Table 2:**
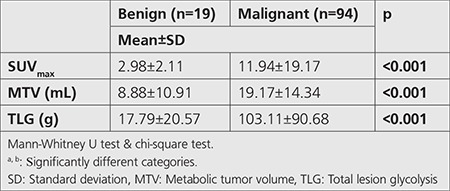
Positron emission tomography/computed tomography findings of benign and malignant solitary pulmonary nodules

**Table 3:**
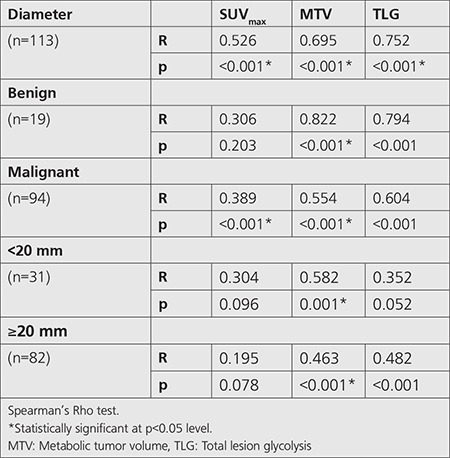
Relationship between ^18^F-FDG positron emission tomography/computed tomography SUV_max_ and volumetric parameters and diameter

**Table 4:**
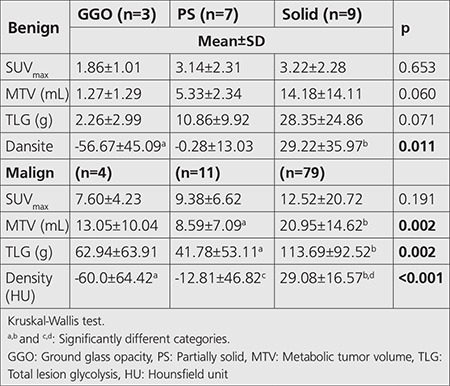
^18^F-FDG positron emission tomography/computed tomography SUV_max_ and volumetric parameters according to attenuation classification

**Table 5:**
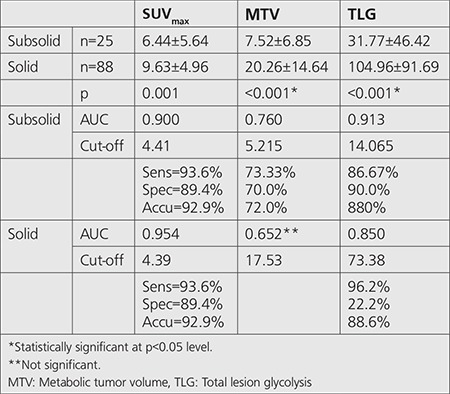
Cut-off values of SUV_max_, metabolic tumor volume, total lesion glycolysis for subsolid and solid solitary pulmonary nodules
